# Industrial metabolism and territorial development of the Maurienne Valley (France)

**DOI:** 10.1007/s10113-021-01845-4

**Published:** 2022-01-11

**Authors:** Magali Talandier, Myriam Donsimoni

**Affiliations:** 1 Grenoble Alps University, UMR PACTE, Institut Universitaire de France, 14 bis avenue Marie Reynoard, Grenoble, France; 2 University of Savoie Mont-Blanc, UMR PACTE, 25 rue Marcoz, Chambéry, France

**Keywords:** Territorial capability, Metabolism, Territorial economics, Territorial ecology, Socio-economic flows

## Abstract

The globalisation of exchanges has resulted in excessive growth of material and immaterial flows. The disconnection among the supply, production, decision-making and consumption sites generates new spatial interdependencies. It determines local socio-economic dynamics and affects ecosystems. In this context, the question arises if territorial capability—“localized collective capacity to serve territorial development”—influences, from local level, these globalised flows systems. By combining territorial economic principles and territorial ecology approaches, we study the industrial metabolism of the Maurienne valley in France. The Maurienne case shows how territorial characteristics contribute to the economic resilience in rural areas. The calculation of wealth flows provides information on the local economic base, the weight of industry and its social impact. The analysis of physical flows reveals the materiality of this industry and the dependence on external resources and international companies. It highlights the various pressures and risks on the environment. To deal with these constraints, companies rely on relational and geographic proximities with local subcontractors. These relationships determine both the proper functioning of the local industrial system and the territorial capability to maintain and transform industrial activities. Most rural European territories experience the same industrial issues and environmental challenges. Therefore, this study offers new research perspectives to better understand and promote ecological transition in old and often rural industrial areas.

## Introduction

The interactions between nature and economic activities have resulted in industrial systems that generate increasingly globalised flows (Storper 2004) of materials (incoming and outgoing flows of raw materials and commodities) and services (circulation of wealth, information and knowledge and the expansion of company networks and partnerships, etc.). The excessive growth of these flows has gradually resulted in a greater distance between production sites and decision-making sites, as well as consumption sites. This spatial separation indicates the need to redesign socio-economic mechanisms, particularly through metropolisation (Krugman 1991) while considering the need for distancing from the environmental consequences of the economic development of a city or region. In this context of trade globalisation and its environmental impacts, many authors and experts consider the local level to be a relevant scale for raising awareness and acting in favour of an ecological transition (Pesch et al. 2019; Coenen et al. 2012; Hansen and Coenen 2015; Hopkins 2008). However, are territories capable of utilising these globalised flows on which their development depends? The use of the concept of territorial capability can help us provide some answers.

The concept of capability was first introduced at the individual level (Sen 1980). The capability approach purports that freedom is based on what people can do in the pursuit of their well-being, thus indicating the kind of life they are able to lead (Robeyns 2016). The same idea can be applied at a territorial level. We consider that the quantity of resources available in a territory is not sufficient to explain its development. The most important factor is what the actors are able to do with these resources. This echoes Sen’s notion of “positional objectivity” to show that objectivity can vary depending on the space in which it expresses itself, without necessarily losing its objective character. Territorial capability would be this objectivity, this local logic that builds the territory and specifies its development. (Andersen and Bøllingtoft 2011; Sassen 2012; Dissart 2012; Lessmann and Rauschmayer 2014). It stems from territorial intelligence that frees up collective action and supports the desire of a group of local stakeholders to act and react (Soula 2017). Territorial capability is defined as a localised collective capability (Buclet and Donsimoni 2018) that is best analysed according to territorial resources (social and natural resources) and how they are used. Sen (1992) associates this aspect with the idea of “functioning”. “Functionings” are the various states of being or doing, i.e. what an individual (or territory) does with the goods (or resources) available to them. At the level of a territory, a trade-off must be made between the maintenance of various economic and social activities and their impacts on the environment (land use, water resource availability), which have significant consequences. However, in a globalised world, this type of arbitration does not depend only on local actors. This is why we propose considering territorial capability as a localised collective capability to act on local resources in a system of multi-scale flows. These flows are constitutive of territories and determine their development dynamics (Davezies 2008; Crevoisier and Rime 2020). This is why territorial capability depends on the collective capacity of a territory to act on material, immaterial and social flows by relying on the resources at its disposal. Here, the territory is not only an institutional area, but it also becomes a dynamic organisation that takes full control of its growth trajectory, either by seizing an opportunity or bypassing an obstacle (Buclet and Donsimoni 2018) but always in cooperation with other territories. An analysis of these interdependencies, from the hinterland to more far-off exchanges, provides better knowledge about levers for local action (Barles 2012; Bahers et al. 2020). Understanding this territorial capability of utilising multi-scale flow systems is key to tackling major environmental challenges and to getting territories to commit to an ecological transition.

Thus, an analysis of territorial capabilities must be based both on the study of material and social flows generated by the territory and on its system of stakeholders. In regional and urban studies, two main approaches consider these spatial interdependencies arising from a territory, territorial economics and territorial ecology.

The territorial economics focuses on the flows of income generated and attracted by the territory, as well as on the stakeholders and populations from which they originate. These studies use the concept of the revisited economic base model (Talandier & Davezies 2009; Segessemann and Crevoisier 2016; Ruault 2018; Crevoisier and Rime 2020). These authors analyse the mechanisms by which wealth circulates. They factor in the social redistribution of territorial economic systems but do not consider the materiality and environmental impact of these flows. In contrast, territorial ecology is based on the concept of metabolism, which reveals the materiality of these flows. However, the social aspects in the sense of the impact on household income are not discussed here.

All these interdependent systems characterise the territories and their capacity for action. By reconciling the approach of territorial economics and territorial ecology, we propose to analyse the industrial system of the Maurienne valley, both in its material and social dimensions. The social dimension includes income flows and local cooperation.

While territorial economics focuses on the economic development of urban and rural areas, most metabolism studies are on cities or regions. We know that the economic weight and the material and energy weight of cities are decisive for the future of the planet. According to the “UN Environment” and the “International Resource Panel”, cities are responsible for approximately 80% of energy consumption, 40 billion tons of material consumed and 65% of air emissions (Swilling et al. 2018). However, the study of these mechanisms in rural areas is of great importance for at least two reasons. First, these are extremely fragile areas. They are home to natural and ecological resources under significant pressure, both on an anthropogenic scale and due to climate change. As such, they are laboratories in which the rapid transformation of environments can be observed. Furthermore, they are the cities’ supply areas. The hinterland provides materials, manufactured goods, wood for various industrial uses and services, which are transported by lorry, freight and maritime transport via port infrastructures (Hall and Hesse 2013). These interdependencies show that the durability of systems cannot only be considered at the city scale. However, most studies do not address what occurs outside of urban borders (Bahers et al. 2020). Thus, according to Bahers et al. 2020, without the “territorialisation” of urban metabolism, the city would remain an unopened “black box” (Huang et al. 2018) without any real contextualisation. In addition, as reminded by these authors, Neil Brenner (Brenner 2014) insists on the existence of environmental injustices between people and places (Brenner 2009) that underlie urban metabolism policies. Brenner believes that urban actors control all resources. In this context, do rural areas still have the capability to act on their resources?

This article aims to analyse the material and social flows linked to the rural industry of a French valley. This analysis of the different economic systems reveals territorial capabilities. This article is an outcome of collective research carried out within the “Cross Disciplinary Project named Trajectoires”, financed by the Idex programme of the French State.

## Territorial economics and ecology: framework and methods

The aim of the method used is to enable a joint study of social and material flows in a rural territory, namely, the Maurienne valley. The study is based on a quantitative and exhaustive analysis of the Maurienne valley’s income flows with an aim to examine its socio-economic system, according to the territorial economic approach. In addition, we develop a qualitative analysis of the spatial interdependencies generated by material and energy flows and stakeholders involved in industry in the Maurienne valley, according to the territorial ecology approach.

### Quantitative analysis of income flows according to territorial economics

In the territorial economics, the analysis of income flows is based on the concept of the economic base model, which has been revisited by regional economists over the last decade (De Keermaecker et al. 2007; Talandier & Davezies 2009; Guimarães et al. 2014; Nesse 2014; Segessemann and Crevoisier 2016; Ruault 2018; Crevoisier and Rime 2020). These approaches distinguish the economic base of a territory, made up of income attracted from the outside, from the non-basic (or domestic) sector, which circulates this income locally within the territory studied.

**Fig. 1:**
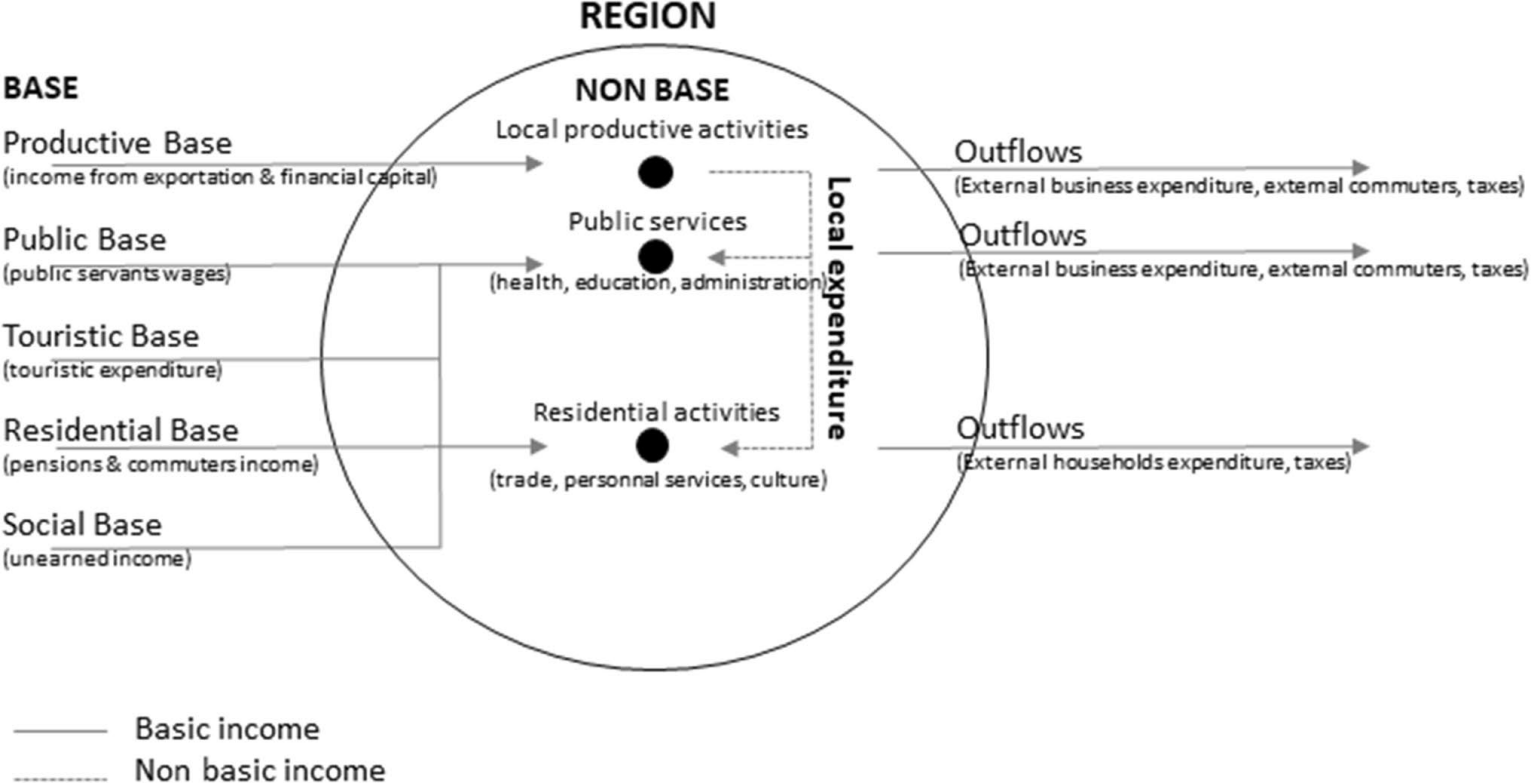
Circular flows of income in the revised economic base theory

Figure 1 summarises the conceptual framework. The economic base is the driver of local development. Five types of bases can be distinguished:

- The productive base is made up of all income from activities involving the production of goods and services to be exported.
- The public base is comprised of the salaries of public-sector employees paid by the French state.
- The tourist base contains all tourist expenditure.
- The residential base is made up of income imported through retirees and people who reside within the territory but work elsewhere.
- The social base contains all social benefits and healthcare reimbursements.

This external income then feeds the activities consumed locally, either by local businesses (local productive activities) or by households (residential activities).

According to this model, the economic development of a territory depends on competitive issues relating to the production and export of goods and services as well as the ability to appeal to other sources of private income (tourism, commuters) or income from public redistribution.

The databases used to calculate flows of income are specified in annex 2. Some data is directly allocated to a type of base. For example, the pensions and salaries of commuters are known and tied to the residential base. Tourist expenditure is added to the tourist base. Social income and healthcare reimbursements make up the social and health base. However, income from the working population must be separated from income from basic activities (external) or non-basic activities (local). This distinction is widely discussed in the literature on economic base theory. We generally distinguish between direct methods based on studies on the population or on companies and indirect methods based on statistical indicators. In the second case, several approaches can be adopted: a priori or ad hoc methods, location quotients, required minimum methods, econometric models or histogram methods (Richardson 1985; Poffet 1989; Vollet and Bousset 2002). In practice, the first two methods are the most commonly used. The ad hoc method simply consists of defining a priori whether the sector of activity is basic or non-basic. The location coefficient is more precise and compares each territory’s economic structure with an (often national) average baseline. Many articles present and discuss these approaches (Vollet and Bousset 2002; Ruault 2018). As such, according to these authors, an over-specialisation of the territory in a given sector indicates that the activity is either partially or totally exported. In the first case, up to the national average, jobs in this sector are considered allocated to satisfying local needs; beyond that, they are allocated to the export base. In the second case, all jobs in the sector are considered basic.

Most recent European research is based on a combination of the ad hoc method and the calculation of specialisation indicators. We use the method developed by Davezies 2008; Carlier et al. 2006; De Keermaecker et al. 2007; Talandier and Davezies 2009; Segessemann and Crevoisier 2016; Ruault 2018.

First, we determine which activities pose no issue. For example, manufacturing sectors are basic because the goods produced are exported. Retail trade, current health and human services are non-basic and consumed locally by households (e.g. bakeries, hairdressers, doctors). However, a certain number of sectors require the use of a specialisation rate calculated on the basis of the population. Thus, when the number of jobs per inhabitant in a given sector is higher than the national average, the income from these “additional” jobs is basic; otherwise, it is non-basic.

When calculated in this way, flows of income offer information on the respective weight of different economic development drivers in the Maurienne valley and on redistribution mechanisms. Nevertheless, these analyses do not consider the materiality of the local economic system. To do so, this quantitative approach combines qualitative interviews conducted more specifically with the large industrial companies in the valley.

### Qualitative analysis of flows of materials and of the system of stakeholders according to territorial ecology

Derived from urban ecology Wolman 1965; D and industrial ecology (Kneese et al. 1970; Ayres 1978), territorial ecology aims to describe, analyse and transform the metabolism of territories. This concept refers to the material representation of flows created by interactions between a society and its environment (Barles 2014). It favours an approach by territory, rather than by sector or product, to reveal the different representations and practices of territories (Buclet et al. 2015). It also questions the challenges of dematerialisation and their consequences in terms of governance of these flows (Barles 2014, 2017; Buclet et al. 2015). The authors rely on the concept of territorial metabolism, which includes the analysis of material and energy flows. Moreover, territorial ecology considers that metabolism is not only material (including energy). It is embedded in social, political, cultural and technical processes. Consequently, territorial ecology proposes an expansion of the studies on physical flows to include social flows (Broto et al. 2012; Princetl 2012; Buclet et al. 2015).

In terms of method, analyses of material flows have developed at national and city scales based on the “economy-wide material flow accounts” method (EWMFA). Methods dedicated to intermediate scales (i.e. territorial scale) developed later. Barles’ research in the Île-de-France region (2009) completed by her work in Bourgogne and in the Midi-Pyrénées region (Barles 2014) adapted the European method to French “départements” and “regions”.

In urban or regional contexts, energy flow analysis (EFA) is based on the same principles as material flow analysis (MFA), which is the most commonly used method to quantify metabolism (Kennedy et al. 2011). EFA focuses solely on energy flows of social or industrial metabolism. Several researchers developed EFA for many cities, including Paris (Kim and Barles 2012), Vienna (Krausmann 2013), Beijing (Zhang et al. 2014), Brussels, Milan and Cape Town (Athanassiadis et al. 2017). More generally, tests show a recent surge in scientific studies on metabolism, particularly urban metabolism (Cui 2018; Dijst et al 2018; John et al. 2019; Rosales Carreón and Worrell 2018; Zhang et al. 2015), with a spike since 2010 (Kennedy 2016). In fact, Beloin-Saint-Pierre et al. (2016) provide a list of over 150 urban metabolism studies: from Brussels (Duvigneaud and Denaeyer-De Smet 1975) to Lisbon (Rosado et al. 2014), Buenos Aires, Sao Paulo (Hoornweg and Bhada-Tata 2012) or Paris and the Île-de-France region (Barles 2009). These analyses reveal the pressure exerted by a society on its environment; they measure energy and material performance and the intensity of exchanges and highlight the interdependencies of cities with the hinterland (Billen et al. 2012).

In most sub-national studies, quantitative data are used. However, these accounting methods are impossible to implement on the scale of a rural area such as the Maurienne valley. Many French industrial companies are located in small towns and rural areas. It is important to be able to reveal the materiality of these territories as well to raise the awareness of local public authorities. To do this, we would need data available on a communal scale, which does not exist. As in other studies in territorial ecology (Buclet et al. 2015; Herbelin 2018; Bahers and Giacchè, 2019) or in the field of political-industrial ecology (Cousins and Newell 2015; Deutz et al. 2017), we chose to conduct semi-structured interviews to gain an understanding of the nature, geography and governance of the valley’s material flows. This qualitative method allows the analysis of non-urban, infra-regional or infra-departmental contexts. This corresponds to the local inter-municipalities scope of action. The interviews show us the main material and energy flows produced by the company and the more social aspects of metabolism through the study of local cooperation and subcontracting chains.

We conducted twenty semi-structured interviews: seventeen in major industrial companies, two with public economic development officers and one with the president of the valley’s public management authority. A list of individuals interviewed is provided in the table in annex 3. In total, the companies interviewed accounted for over 80% of industrial jobs in the valley. The questions asked were of an operational and strategic nature. We focused on respondents who were managers, CEOs and heads of departments. We conducted the interviews in 2018 based on a semi-structured format with three main topics and twelve sub-questions (see questionnaire in annex 4). The three main themes were:

- The company’s characteristics (sector of activity, number of jobs, head office, historical presence, main partners or competitors);
- Material flows, access to resources (the import and export of materials, products, mobilised resources, logistical requirements, energy requirements, ties to the valley)
- Relations with other establishments and anchoring in the valley (subcontracting, partnerships, benefits and limits of being located in the Maurienne)

We have transcribed all interviews into text.

We used information from interviews to recreate and map the flows of materials and inter-company relations generated by the valley’s industries. Although the objective was not to precisely quantify biophysical data (whether primary or secondary), when the respondents had information on the volumes of these flows, we provided details of them.

Our results are therefore based on an approach that studies rural areas:

- Flows of income to consider the economic drivers and the social impact of industry.

- The physical flows to reveal the materiality of industry.

- The stakeholder systems through the subcontracting chain.

These elements elucidate the spatial interdependencies, from local to global aspects, created by the valley’s industrial system. This territorial systemic approach should help us better understand territorial capability to enhance and protect its resources.

## Case study: the Maurienne valley’s socio-economic system

The Maurienne valley is a rural territory located in the département of Savoie in the French Alps. Spanning 120 km between Italy and the Hautes-Alpes, it is the longest alpine valley and is home to 44,000 year-round inhabitants spread over approximately fifty municipalities. Known for its ski resorts and natural parks, the valley’s economy has multiple sources. Economic life is based on touristic, commercial, logistic and industrial sectors. Since the nineteenth century, Maurienne industries have exploited the hydroelectric resources of the arc that runs through it. Initially exploited by the paper industry, white coal rapidly attracted the electrochemistry and electrometallurgy sectors. Industrialists converted, developed and managed the valley’s hydroelectric network until 1946, when electricity was nationalised in France. Today, EDF (Electricité de France) operates 17 power plants and 4 major dams in the Maurienne valley, representing 10% of the peak load across the entire French hydroelectric fleet (i.e. 4 billion kW produced per year with an installed capacity of 2.43 GigaW). With its negotiated tariffs, this abundant hydroelectric resource is one of the valley’s main industrial assets. However, since the 1970s, the industrial sector has been significantly restructured, and the number of jobs has been halved. Today, Maurienne’s industrial past survives thanks to four large companies: Trimet, Ferropem, Arkema and Onera. Trimet is the current name (since 2013) of the former Péchiney factory, renamed Rio-Tinto Alcan in 2008; it is the valley’s industrial jewel. With approximately 600 employees, Trimet is the leading industrial employer and the valley’s second employer after the Saint-Jean-de-Maurienne hospital. This aluminium company also provides the territory with an estimated 1800 jobs, i.e. nearly 7% of all jobs in the valley. Significant investments (in association with EDF for 35% of capital) have enabled the replacement of electrolysis cells and have increased aluminium production to 150,000 t/year, i.e. 40% of national production in 2014, with the Dunkerque port site providing the remaining 60%. The Montrichet Ferropem factory, a subsidiary of the FerroGlobe group, still specialises in high-purity silicon, a promising market since the 2010s with the development of photovoltaic technology. It produces 35,000 tons of silicon per year, 10% of the group’s production. It employs 150 people. The Arkema company occupies a leading position in the solvent and amine markets. Amine production on-site has developed and diversified into special products with higher added value that are more angled towards the pharmaceutical and cosmetic industries. The company employs 160 people. ONERA is the French centre for aerospace research; it is under the supervision of the ministry of defence and has a fleet of wind tunnels representing over half of the European fleet. Approximately 160 people currently work there.

In total, these four companies account for 1000 industrial jobs. In line with these export activities, there are currently 80–90 companies acting as subcontractors for these large groups in the valley. All of these establishments employ approximately 2800 people, representing 21% of total employment in the valley.

In addition to tourism, the industrial economy is a major lever for local socio-economic development. Exposed to numerous socio-economic (deindustrialisation) and ecological (depletion of resources) shocks and uncertainties, the territory’s capability of managing its industrial trajectory is in question.

The results are drawn from the comprehensive quantitative analysis of the valley’s flows of income, combined with the qualitative analysis of material flows and stakeholder systems in the industrial sector.

**Fig. 2:**
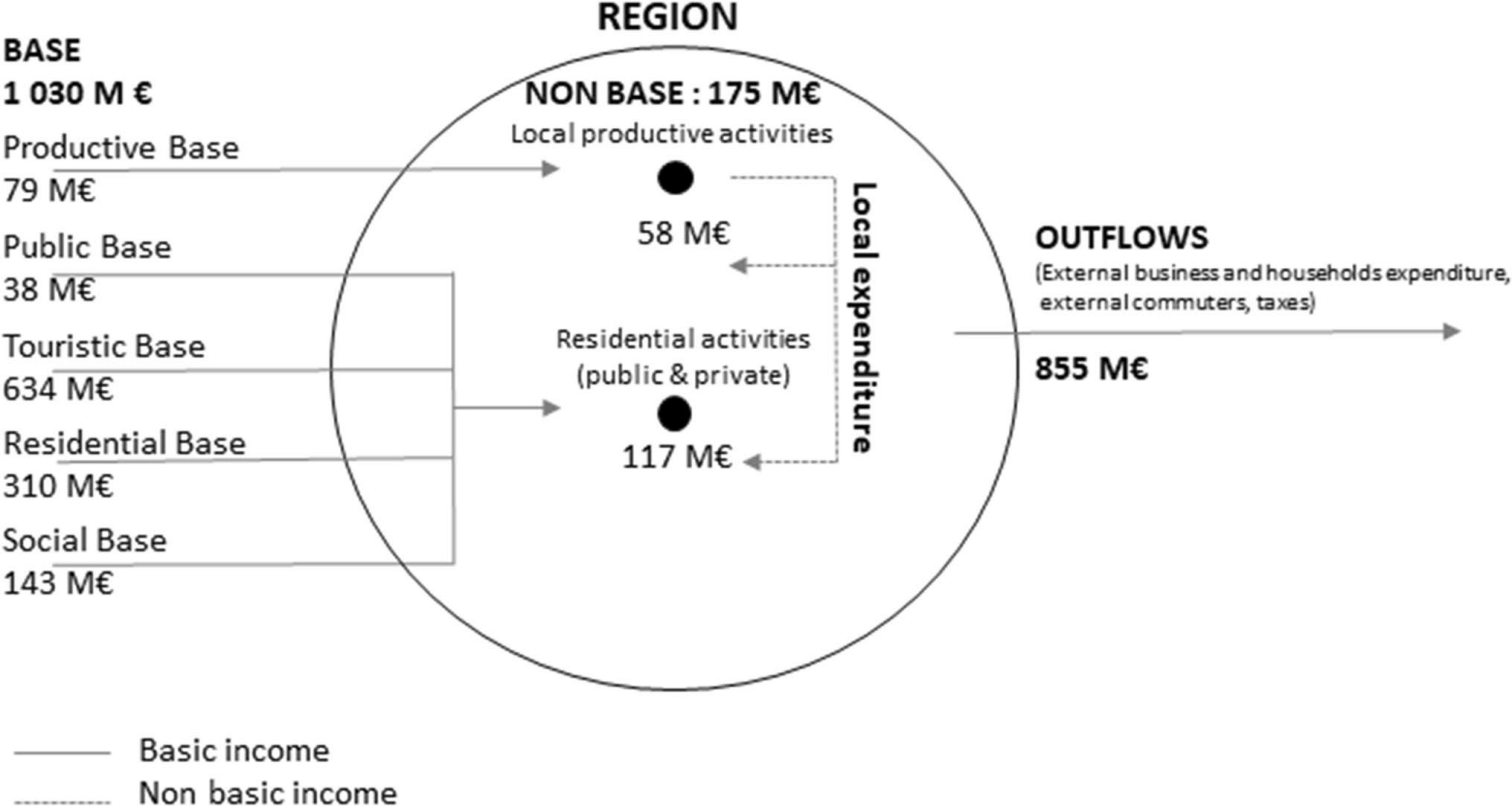
Circulation of flows of income in the Maurienne valley (in millions of €, M€)

Figure 2 provides the results for the Maurienne valley, based on 2014/2015 data, in millions of euros.

In total, the valley’s economic base represents approximately one billion euros and is made up of different types of income. The results underline the weight of external flows of income (base and outflows) compared to internal flows. Thus, the Maurienne valley’s socio-economic system is largely based on flows of income attracted from outside of the valley due to production, tourist, residential and public factors.

Tourism clearly dominates the dynamics of the valley’s economic development. Two-thirds of the economic base is the result of tourist expenditure (€634 M). A portion of this expenditure will leave the territory in the form of remuneration for financial transactions, seasonal workers, duties, taxes, etc. We also know that tourist jobs are growing quickly (+ 18% between 2007 and 2013), which is not the case in the valley’s other economic sectors, e.g. − 21% for industry and − 13% for the construction industry over the same period. Thus, we are witnessing a hyper-specialisation of the Maurienne valley’s economy centred on tourism. The residential base is also an important source of external income, both through pensions paid out (€185 M) and commuters (€125 M) living in the valley but working elsewhere. These non-industrial bases are complemented by income from the public (€38 M) and social redistribution (€143 M). Ultimately, productive export activities now represent a very low proportion of the economic base attracted by the valley, i.e. €79 M, or 7% of the economic base.

If spent in the valley, these sources of income lead to the development of local, productive and residential activities. These are activities introduced in the valley, either by companies or by households. In total, approximately 17% of basic income (i.e. €175 M of €1,030 M) is converted into income for workers in non-basic sectors. The local production sector represents €58 M in income every year, and the local residential sector represents €117 M.

The following section of the article focuses on the flows of materials and relations between economic stakeholders created by the valley’s industrial activities. These activities are the source of nearly all local productive bases, which generate €137 M in income per year. The interviews conducted with large industrial companies now enable us to determine which material and organisational interdependencies are created by these flows of income to better determine the territory’s capability of utilising its industrial system.

### System of flows of materials and relations between industrial stakeholders

The results of the interviews conducted with CEOs and executives from industrial companies in the valley allow us to distinguish between different scales of spatial interdependency, from material international exchanges to local services. These spatial interdependencies depend on the nature of the relations observed. The first map (Fig. 4) summarises only flows of imported and exported materials. It highlights the valley’s interdependence in a globalised market. The second map illustrates the power ties between the valley’s large companies, both through inter-industry relations within the valley and their relations with their head office, which are usually located in Europe (Fig. 5). Last, the third map highlights the business service flows generated by these large industrial companies at the local level within the valley itself (Fig. 6).

**Fig. 3:**
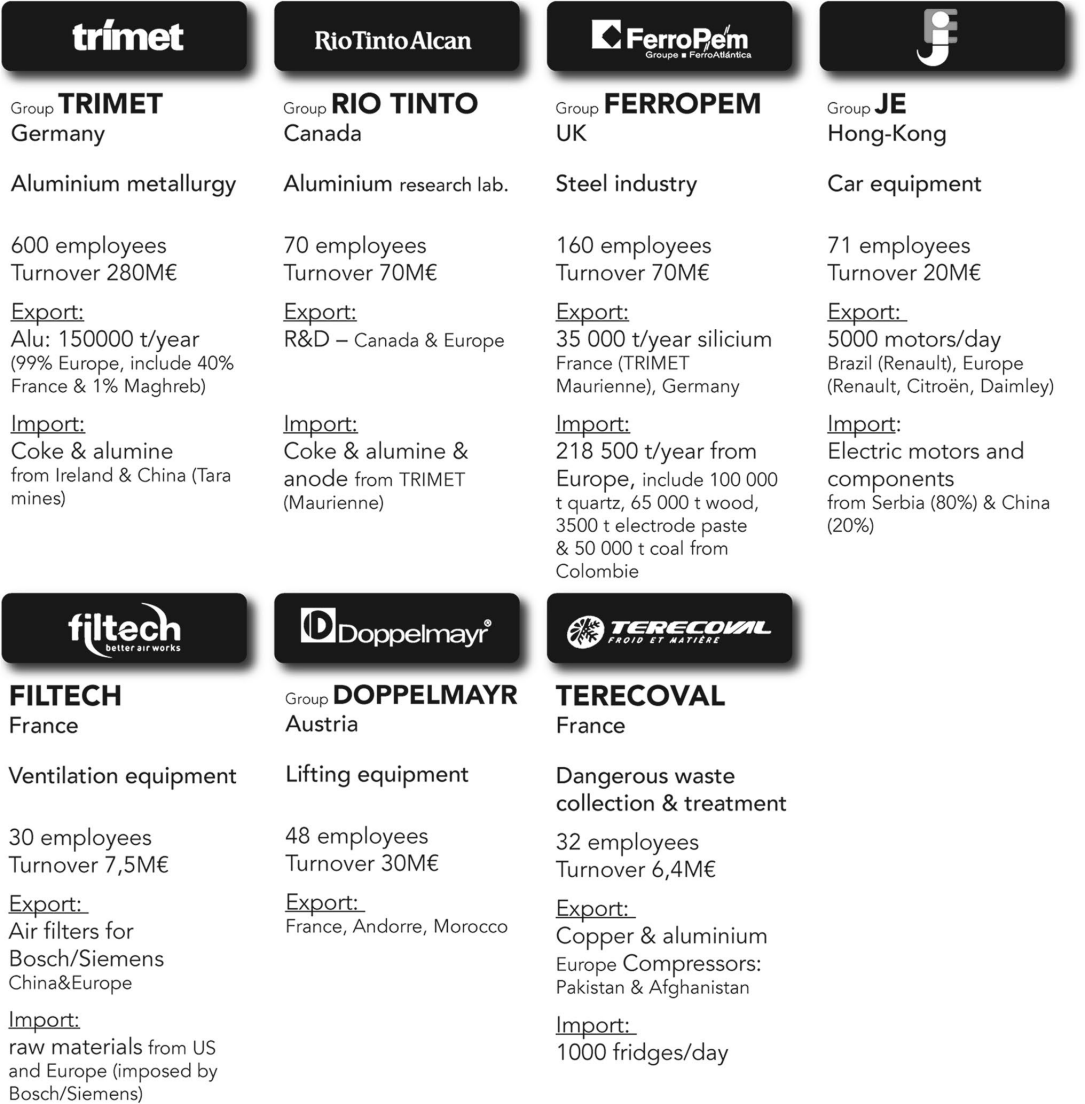
Characteristics of industrial large enterprises

**Fig. 4:**
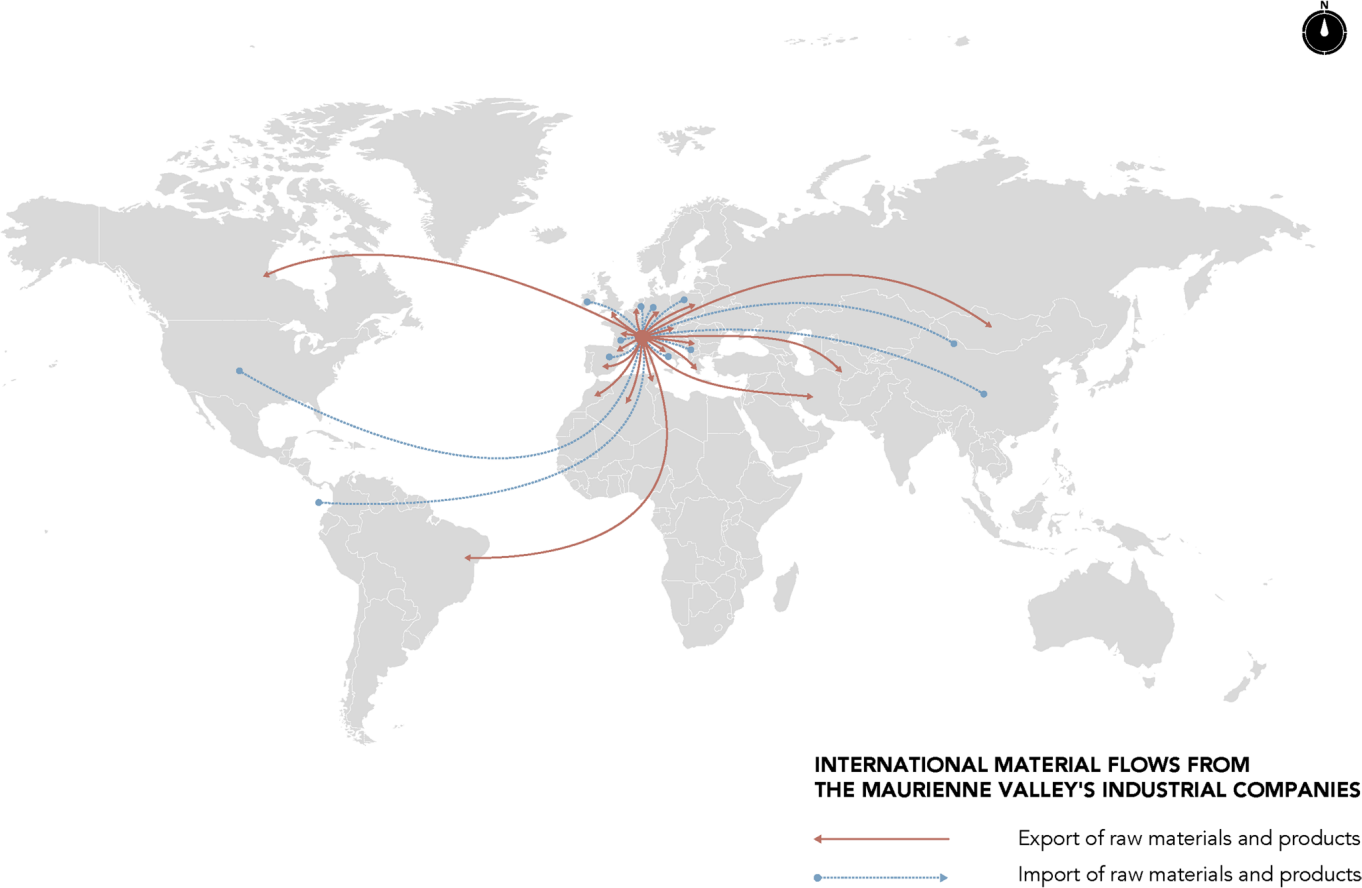
Map of materials international flows

**Fig. 5:**
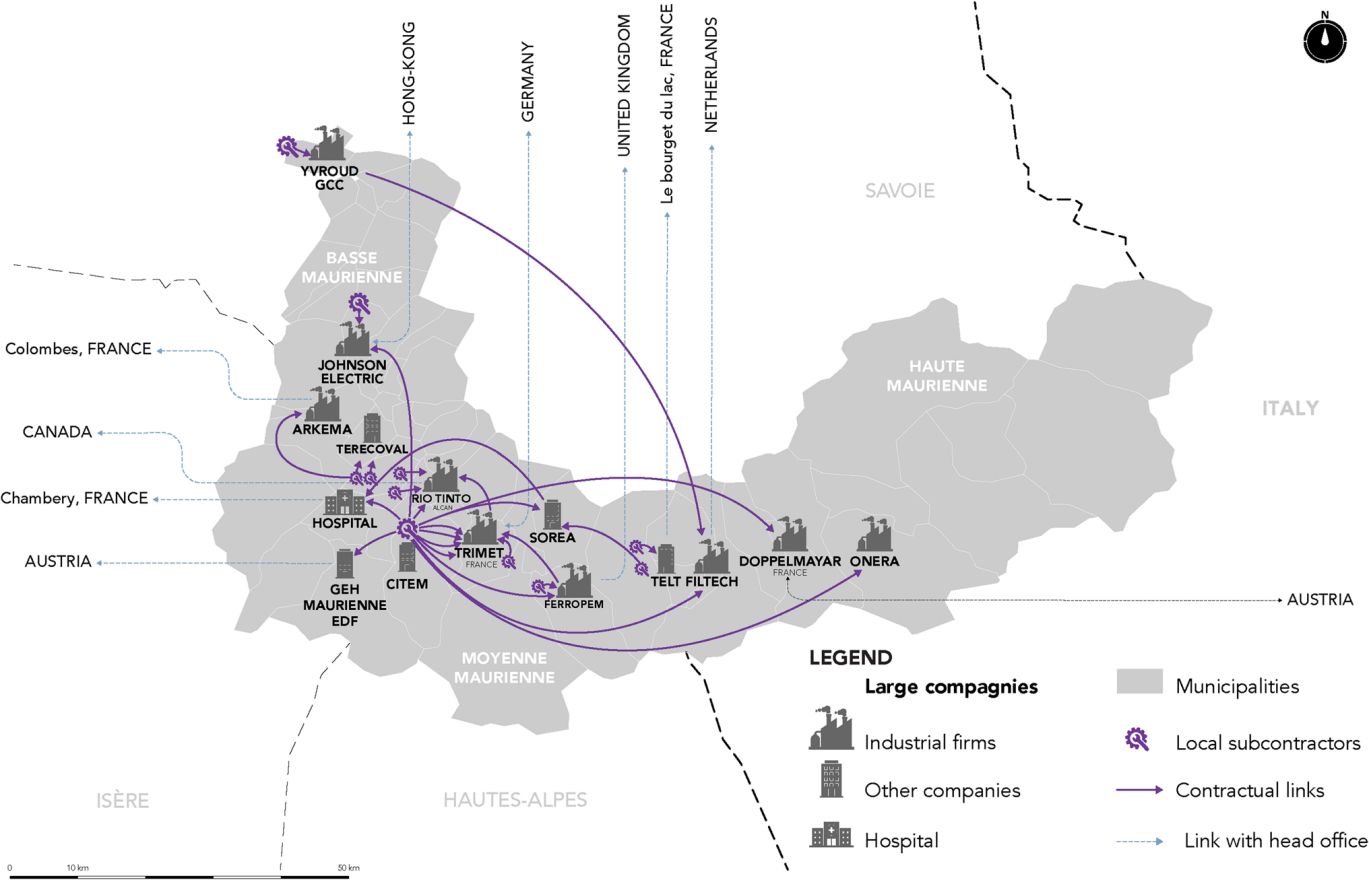
International and local network of the Maurienne valley’s large companies

Figures 3 and 4 shows the influence of the flow of materials that are imported and exported by the valley’s large industrial companies. Several observations can be made. First, the Maurienne valley’s industrial activity is fully globalised. All raw materials are imported from all continents, and all production is exported on an international scale. While this inclusion in globalisation does promote Maurienne knowledge at the global level, it also begs the question of its capability of utilising these flows. For example, (more details in annex 5), Trimet—the valley’s flagship company—receives its supplies (250,000 tons of alumina/year, 70,000 tons of coke and cryolite from Ireland) by full trains from Fos-sur-mer. A specific train line (the TRIMET Express) transports raw materials with three rotations of 66 wagons per week from Fos-sur-mer in collaboration with SNCF (national railway operator). The valley’s other large companies deliver their raw materials by lorries from all over France and abroad. The same applies for the valley’s manufacturing exports.

The map of cooperation between the valley’s large companies confirms that there is no real local partnership system (Fig. 5). Of the valley’s 15 industrial companies, only two maintain customer-to-supplier relations, thereby creating material flows within the valley. Johnson Electric purchases plastic parts from Jemaplast, the main components for the manufacturing of its fan units. Trimet entertains close ties with LRF Rio Tinto Alcan (historically from the same company), which conducts R&D on technologies relating to the operation of electrolysis cells and provides Trimet with liquid aluminium to manufacture its ingots and wires. Trimet supplies LRF Rio Tinto Alcan with alumina and coke.

Furthermore, from a decision-making point of view, the head offices of most of these companies are located in other European countries, Canada, Australia or even Hong Kong.

**Fig. 6:**
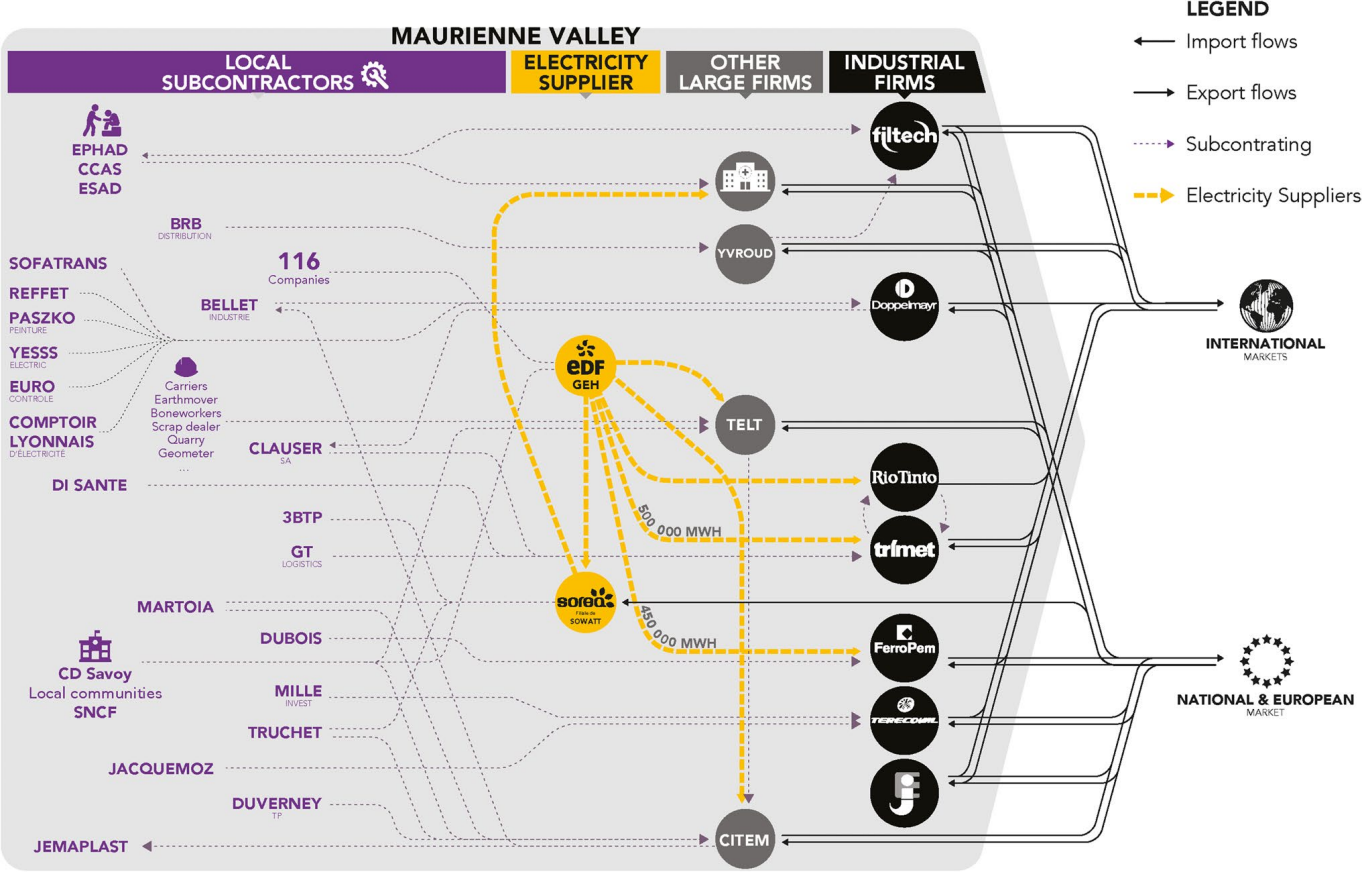
Local network of subcontracting by the Maurienne valley’s large companies

Last, Fig. 6 highlights all of the material and contractual relations established by these large companies at the local level. Here, the spatialisation of these flows clearly reveals a “valley” effect. The suppliers of generic materials (electric, office materials, etc.), subcontractor companies for transport, cleaning and maintenance or the disposal of waste are almost exclusively located in the Maurienne valley (Clauser, Di Sante, Jemaplast, etc.). The ripple effect in the other territories in Savoie is very weak. In close proximity, the valley offers most of the services and energy resources required by the large industries studied. All of the large companies interviewed stated that they preferred to use these local partnerships for reasons relating to the transaction cost but also relating to knowledge. Geographical proximity provides the guarantee of creating value in the valley and of ensuring real responsiveness from the companies that know each other well. This proximity is both relational, organisational and spatial (Boschma 2015; Knoben and Oerlemans 2006; Torre and Rallet 2005). Thus, new production activities have developed in collaboration with the valley’s main outsourcers. These micro-enterprises and SMEs currently contribute to boost the local economy. They are essential in understanding and structuring the territory’s capability.

### Discussion on the territorial capability of governing economic flows

In a world of globalised flows of materials and services, a territory’s capability depends on collective intelligence to optimise these spatial interdependencies and overcome external shocks. In this context, what levers for action are available to a rural territory such as the Maurienne valley? The results of the systemic analysis of the valley’s economic flows provide us with answers to this question and items for discussion.

The analysis of flows of materials and contractual relations within the industrial sector allows us to delve deeper into the understanding of the territory’s environmental challenges and capabilities in this respect.

In terms of flows of materials, the valley’s industry is completely dependent on incoming (import of raw materials) and outgoing (export of products) international flows. The Maurienne industry is therefore significantly exposed to external economic (Hassink 2010; Hudson 2010), geopolitical (Habib et al. 2016; Caldara and Lacoviello 2018) and ecological shocks (depletion of the resources used). Therefore, its territorial capability appears quite weak. However, the local environmental impacts of this industry are real, and the industrial risks to valley inhabitants are high (annex 6). In fact, 6 companies in the valley are classed as Seveso^1^ risks. Maurienne is also affected by the transport of hazardous materials, with industrial establishments being supplied via road A43 motorway or by railways and with a gas pipeline passing through the entrance to the valley. Several large dams have been created for hydroelectric production, including two sizeable dams listed on dam failure risk maps: Bissorte and Mont-Cenis. Hydropower is instrumental (2.781 Gwh/year) for the valley. Global warming also affects water and snow resources. A forecast study conducted in 2005 shows that glaciers will have completely disappeared by the end of the century (Zemp et al. 2019; Zekollari et al. 2019). All studies conclude that the availability of water in the Alps will decrease. At the same time, demand is increasing (leisure, industry, energy, agriculture, etc.). Tensions are emerging around the consumption and preservation of this resource.

In the industry, the main advantage of being located in Maurienne remains hydropower. Although original contracts with EDF ended in 2015, large companies continue to benefit from advantageous tariffs through “electricity-intensive” contracts^2^. These companies are supplied with electricity by RTE (an electric transportation network via high-voltage lines). For example, Trimet, a company that consumes the equivalent of a city with 500,000 inhabitants on a daily basis, has entered into an agreement with RTE to “withdraw” from the network when necessary. The valley also has several local electricity supply and network maintenance companies (former municipal electricity undertakings) with semi-public company status (SOREA, SOWATT, etc.) with capital from local municipalities and the private sector. Works on a new hydroelectric plant are currently being completed. By harnessing the waters of the Arvan, a mountain stream running a few kilometres long, this new plant will produce close to 8 million kWh per year from the winter of 2019. More recently, these firms are developing a policy for diversification into the world of renewable energies.

The prevention of industrial risks and the management of hydraulic resources mobilise the valley’s territorial capability. There have been crises, negotiations and arbitrations that have required a type of collective intelligence and a certain resilience capacity against shocks. To increase its resilience, the territory tries to develop other resources at its disposal: solar, wood, geothermal, wind, waste, etc. The territory has the capability to utilise local resources but not the globalised flows of the industry.

The analysis of income flows shows that the Maurienne economy is essentially based on the tourism sector. One might ask why this territory supports an industry that has a heavy impact on its ecosystem. In fact, the research conducted by regional economists shows that a diversified economic structure (Brun and Greenbaum 2016; Crescenzi et al. 2016) is more resilient than a more specialised structure. Different sectors have different sensitivities to changes in companies, export markets, monetary conditions (exchange rate and interest rate), etc. (Martin 2012). In contrast, when an economy is highly specialised, its resilience depends on the nature of its specialisation, as well as the type of crisis experienced. For example, international tourism was a resilient sector during the economic crisis of 2008 but has weakened local economies since the COVID-19 pandemic. Thus, with balanced socio-economic development in mind, local decision-makers and economic development stakeholders stated that they were particularly attentive to maintaining a productive industrial fabric in the valley. In addition to its history and its related identity and cultural aspects, the industry offers an economic, seasonal (multiple activities of mountain workers) and geographic complementarity (industry in the valley and tourism in the mountains). Furthermore, the results show that the valley’s exporting industrial companies have created 80 to 90 subcontracting micro-enterprises and SMEs. Today, the economic weight of these local activities, in terms of income generated, has become almost as important as that of large companies. Moreover, the analysis of flows of income relating to commuter migration shows that 90% of a Maurienne company’s employees live in the valley and therefore potentially spend a large amount of their income in support of local trades and services. In terms of capability, these results underline the importance of exploiting the complementarity of industry and tourism, the two main economic sectors, and maximising the local circulation of wealth (strengthening of local productive and residential activities).

More precisely, the analysis of cooperation and contractual relations generated by the valley’s industry reveals the wealth of the local economic system. The industry doesn’t seem to be anchored to the valley. Maurienne’s large industrial companies have established their head offices abroad, and the relationships between these large companies are considerably weak. Thus, where once, between the world wars, a local productive system had developed around industry, there is now a more hierarchical organisation with the large companies at the top of the pyramid (often acquired by foreign investors). However, the qualitative analysis of subcontracting ties between establishments shows to what extent these large companies rely on the local network of micro-enterprises and SMEs, nearly all of which are located in the valley. The international reputation of some key local companies contributes to using and sustaining local knowledge. As a result, large historical companies remain in the valley. This territorial anchoring is an indisputable element of capability. Subcontractor micro-enterprises and SMEs’ flexibility are necessary to ensure that large companies are responsive and competitive. The company managers we met recognise that subcontractors are an invaluable asset, and that the valley’s industrial resilience relies on their unique expertise. This collaboration builds collective intelligence and improves the territorial capability, but there is still work to be done on the ecological transition. Local authorities have a key role to play in this regard.

## Conclusion

Maurienne’s industry is vital to the valley’s economy. However, the large companies depend on global markets and are vulnerable to external shocks. The pressure on the environment is high, due to numerous threats from both climate change and resource depletion. In this context, the territory’s capability is questioned. It implies collective intelligence to optimise the spatial interdependencies and thus better guard itself against future shocks. The analysis of economic relations has revealed the existence of a prosperous subcontracting system with strong local roots and specific knowledge. The network of micro-enterprises and SMEs, in close collaboration with the valley’s large industries, strengthens the local capability to utilise these resources. If the history and specific details of Maurienne partly explain these results, then its trajectory is not atypical, and it is not an isolated case. Maurienne shows how territorial characteristics are important for resilience. Most rural European territories with an industrial history experience the same deindustrialisation issues (Hobor 2013; Cercleux and Bole 2009) and must address the same types of questions and environmental challenges (Boschma 2015; Giannakis & Bruggeman 2017). Therefore, the conclusions from this study also apply to other European rural industrial areas. By combining territorial economics and territorial ecology concepts and methods, this study offers new research perspectives to better understand these types of territories in their ecological transition.

## Footnotes

1 “Seveso Directive” is one of three European Union Directives to improve the safety of sites containing large quantities of dangerous substances.

2 In France, a company is considered electricity-intensive when its electricity consumption exceeds 2.5 kWh per euro of added value (turnover minus intermediate costs). Based on this criterion, in France, over 500 industrial companies are considered electricity intensive. They mainly operate in three large sectors: the steel industry, the chemical industry and the paper and cardboard industry. They use up approximately 20% of national electricity consumption and employ a total of close of 100,000 employees.
